# Dysregulation of FBW7 in malignant lymphoproliferative disorders

**DOI:** 10.3389/fonc.2022.988138

**Published:** 2022-11-09

**Authors:** Xin Wan, Wei Guo, Zhumei Zhan, Ou Bai

**Affiliations:** Department of Hematology, The First Hospital of Jilin University, Changchun, Jilin, China

**Keywords:** FBW7, lymphoproliferative disorders, ubiquitin, Notch, c-Myc

## Abstract

The ubiquitin-proteasome system (UPS) is involved in various aspects of cell processes, including cell proliferation, differentiation, and cell cycle progression. F-box and WD repeat domain-containing protein 7 (FBW7), as a key component of UPS proteins and a critical tumor suppressor in human cancers, controls proteasome-mediated degradation by ubiquitinating oncoproteins such as c-Myc, Mcl-1, cyclin E, and Notch. It also plays a role in the development of various cancers, including solid and hematological malignancies, such as T-cell acute lymphoblastic leukemia, diffuse large B-cell lymphoma, and multiple myeloma. This comprehensive review emphasizes the functions, substrates, and expression of FBW7 in malignant lymphoproliferative disorders.

## Introduction

Malignant lymphoproliferative disorders are a heterogeneous group of disorders characterized by the abnormal proliferation of lymphocytes, ranging from indolent to highly aggressive neoplasms, including chronic lymphocytic leukemia (CLL), diffuse large B-cell lymphoma (DLBCL), multiple myeloma (MM), and acute lymphoblastic leukemia (ALL). Although targeted drugs have significantly improved the prognosis of patients with malignant lymphoproliferative disorders, a subset of patients have a remarkably poor outcome. Therefore, exploring the underlying mechanisms of malignant lymphoproliferative disorders is critical (1).

F-box and WD repeat domain-containing protein 7 (FBW7), also known as FBXW7, hCDC4, and AGO, is located at chromosome 4q31, which is a genomic region deleted in more than 30% of cancers (2). Oberg and colleagues determined that FBW7’s role in substrate recognition is a key component of the Skp1−Cul1−F−box (SCF)−type E3 ubiquitin ligases (3). FBW7 has three isoforms: FBW7α, FBW7β, and FBW7γ; all of them contain conserved interaction domains of the F-box and WD repeats in the C-terminus. However, their 5’-UTR and N-terminal coding regions are different, forming the specificity of the expression and function of these three protein isoforms. These three isoforms also have distinct cellular localizations. FBW7α is localized in the nucleoplasm, FBW7β is cytoplasmic, and FBW7γ is nucleolar. FBW7α is regulated by protein kinase C and is mainly expressed in mice. FBW7β is beneficial to fight oxidative stress in cells and more expressed in brain tissue. FBW7γ exclusively expressed in skeletal muscle (4, 5).

### Substrates of FBW7 in cancer

FBW7 is an important tumor suppressor in cancer development, and *FBW7* mutations have been detected in a wide range of human malignancies, including lung cancer, ovarian cancer, colorectal cancer, T acute lymphoblastic leukemia (T-ALL), and MM. The percentage of *FBW7* mutations varies across cancers, being up to 20% in T-ALL (2). FBW7 recognizes c-Myc, Mcl-1, and other oncogenic proteins for ubiquitination and subsequent proteasomal degradation, which is closely related to the proliferation, apoptosis, invasion, and metastasis of cancer cells (6–10).

The Myc protein is a transcription factor that has crucial roles in regulating gene expression for cell proliferation, metastasis, and metabolism, and deregulation of the proto-oncogene c-Myc leads to the development of many human cancers, including hematological malignancy such as DLBCL and solid tumors such as gastric carcinoma. The c-Myc protein is ubiquitylated and degraded by the ubiquitin–proteasome system (11–13). FBW7 overexpression results in decreased c-Myc levels, inhibited cell proliferation, and induced apoptosis, while *FBW7* mutations can cause c-Myc accumulation, enhanced cell proliferation, and reduced apoptosis. Thus, FBW7 may mediate apoptosis and growth arrest in leukemia-initiating cells through the ubiquitin–proteasome system and degradation of c-Myc (14).

Another oncoprotein, cyclin E, is a crucial component of the cell cycle and regulates the G1 to S-phase transition and promotes DNA replication. However, cyclin E is frequently dysregulated in cancers, and excess cyclin E activity impairs S-phase progression and causes genomic instability. Significantly, cancers with *FBW7* mutations have increased levels of cyclin E. Furthermore, protein phosphatases, such as PP2A-B56, dephosphorylate Ser384 during the interphase of the cell cycle, resulting in a decrease in FBW7 levels and increase in cyclin E levels (15).

Moreover, the Notch pathway regulates numerous cellular functions, including differentiation, proliferation, and apoptosis. FBW7 plays a crucial role in regulating the Notch pathway, including Notch upstream regulators and downstream substrates. The oncoprotein Notch, which is also as a target for FBW7-mediated ubiquitylation, participates in the development of many human cancers, such as T-ALL and DLBCL (16, 17). Moreover, FBW7 can inhibit metastasis and invasion of CLL cells by regulating signaling pathways, such as Notch and its downstream target molecules (18).

In addition, Mcl-1, as the pro-survival Bcl-2 family member, inhibits apoptosis by blocking cell death in numerous cancers. For example, loss of FBW7 in T-ALL cells increases Mcl-1 expression and promotes chemoresistance (19). The transcription factors nuclear factor kappa B (NF-κB) and c-Jun are also important in the development of lymphoproliferative disorders. NF−κB2/p100 can interact with FBW7 thereby promoting its degradation in a GSK3β phosphorylation-dependent manner (20). In addition to these proteins, FBW7 can also ubiquitylate several essential proteins, such as BRAF (**Table 1**).

**Table 1:** FBW7 substrates and major biological functions in lymphoproliferative disorders.

Gene	Phosphorylationsites	Kinases	Cancers	Biological functions of substrates	References
c-Myc	T58, S62	GSK3	T-ALL, ATL, DLBCL, CLL, MM	Tumor proliferation, metastasis, glycolysis	(11, 12, 18, 21–23)
Notch1	T2512/E2516	CDK8	T-ALL, ATL, DLBCL, CLL,	Notch pathway, proliferation, migration and invasion	(17, 18, 24, 25)
cyclin E	T380/S384, T62/E66	CDK2, GSK3	T-ALL, MM	Cell cycle	(26, 27)
c-Jun	T239/S242	GSK3	MM	Cell proliferation	(23, 28)
Mcl-1	S159/T163 S121	GSK3	T-ALL, DLBCL, PEL, MM	Inhibit apoptosis	(19, 22, 29–31)
NF-κB	ND	GSK3	T-ALL, MM	Cell proliferation, inhibit apoptosis	(20, 32, 33)
BRAF	ND	ND	ATL	BET inhibitors resistance	(34)

ATL, adult T cell leukemia/lymphoma; T-ALL, T cell acute lymphoblastic leukemia; DLBCL, diffuse large B-cell lymphoma; CLL, chronic lymphocytic leukemia; MM, multiple myeloma; PEL, primary effusion lymphoma.

### Regulation of FBW7

Mutations or deletions of *FBW7* have been implicated poor prognosis, indicating that aberrant regulation of FBW7 is one of the factors for cancer progression. Some of the regulators of FBW7 include p53, Numb, microRNAs (miRNAs), and CCAAT/enhancer binding protein-δ (C/EBP δ) (35). P53 is a well-known tumor suppressor protein that conserves genome stability after DNA damage. Moreover, dysregulation of p53 is associated with the development of various cancers. Inactivation of FBW7 could lead to premature loss of hematopoietic stem cells and impaired regulation of cell apoptosis by p53 (36). Another transcription factor, C/EBP δ, decreases Notch intracellular domain (NICD) degradation by ubiquitination through inhibition of FBW7 expression and promotes Notch1 mRNA expression in cancer cells (37). Furthermore, miRNAs, such as miR-223, can regulate T-ALL proliferation by reducing FBW7 expression and contributing to drug resistance (32). In addition, miR-27 and miR-214 could also decrease FBW7 expression to regulate cell proliferation in other lymphoproliferative disorders. However, the exact function of FBW7 in malignant lymphoproliferative disorders remains unclear. Understanding the regulation network surrounding FBW7 is crucial for providing insights into the mechanisms of FBW7-mediated malignant lymphoproliferative disorders. In this review, we focus on the role of FBW7 in malignant lymphoproliferative disorders to identify a novel target for future therapies.

## Proliferation of T lymphocytes

### T lymphoblastic lymphoma/acute lymphoblastic leukemia

T lymphoblastic lymphoma/acute lymphoblastic leukemia (T-LBL/ALL) is a highly aggressive hematologic malignancy caused by the malignant transformation of T−cell progenitors. T−ALL accounts for 15% and 25% of the total number of childhood and adult cases of ALL, respectively. Adults with T−ALL have poor long-term survival. Approximately 50% of them develop recurrence within 1 year, and the remission rate of second-line chemotherapy is only 30%–45%, which ultimately develops refractory leukemia (38, 39). The 5-year survival rate is 10%; however, in patients with recurrent or refractory T-ALL, overall survival (OS) is less than 6 months (40). Therefore, understanding the underlying mechanisms of T−ALL development is critical.

*Notch1* is the most common oncogene for T-ALL, and mutations in *Notch1* have been reported in more than 60% of T-ALL. Besides, the mutation rate of *Notch1 in* adults is higher than children. Mutations of *Notch1* occur mainly in the heterodimeric domain (HD) region and the proline, glutamine, serine, and threonine domain (PEST). Mutations in the HD domain can result in Notch1 activation by reducing the interaction of the Notch1 subunit. The PEST region regulates protein turnover by targeting proteins to ubiquitin–proteasome complex for subsequent degradation (16, 41). The cross-talk between Notch and NF-κB pathways is vital in T-ALL development, indicating NF-κB signaling is one of the major mediators of Notch-induced oncogenic transformation (42). The activation of the Notch pathway can upregulate NF-κB activity, resulting in abnormal cell proliferation and apoptosis inhibition (42). In addition, the occurrence of T-ALL is also related to the abnormal expression of noncoding regulatory elements and miRNAs, such as miR-19, miR-155, and miR-233 (43).

**Figure 1:**
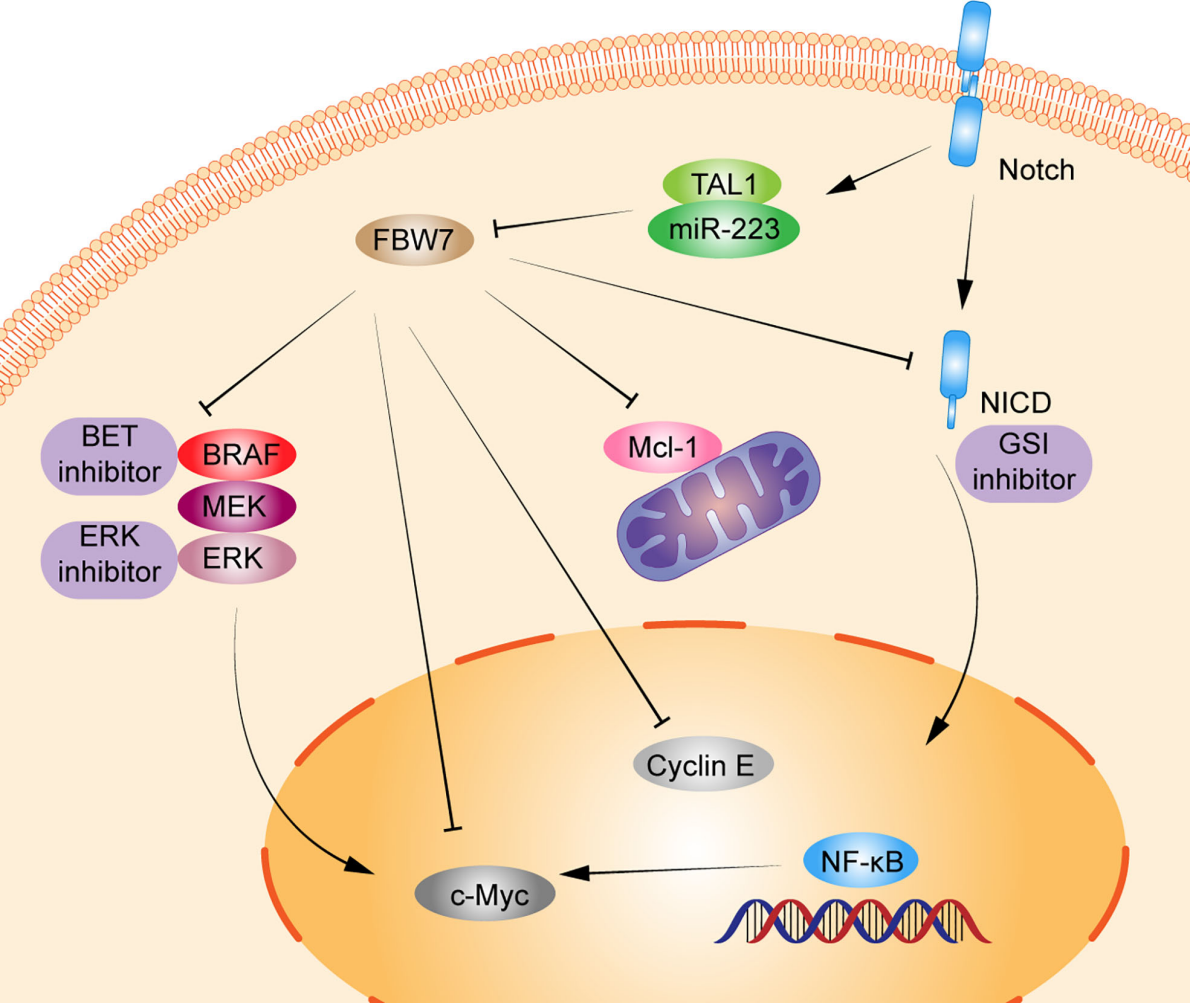
Schematic diagram of FBW7-mediated oncogene and signaling pathway in T lymphoproliferative malignancies. 1) Dysregulation of FBW7 can reduce cellular levels of Notch intracellular Domain (NICD) degradation, which leads to abnormal activation of Notch pathway, NF-κB pathway, and affect the expression of its downstream substrates c-Myc, cyclin E. 2) T-cell acute lymphocytic leukemia 1 (TAL1) can upregulate miR-223 expression, thus reducing the expression of FBW7 and increasing the expression of downstream substrates c-Myc, Notch1, cyclin E, and Mcl-1 to induce cell proliferation. 3) FBW7 mutations activate the RAF-MEK-ERK pathway, inhibiting BRAF degradation and providing resistance to BET inhibitors.

As early as more than 10 years ago, studies have shown that inactivation of FBW7 could lead to premature loss of hematopoietic stem cells caused by an active cell cycle and impaired regulation of cell apoptosis by p53 (36). FBW7 can regulate the cell cycle in a differentiation-dependent manner and loss leads to the accumulation of c-Myc, which results in the excessive proliferation of immature T cells, ultimately leading to the development of T-ALL or lymphoma (44). The mutation rate of *FBW7* in T-ALL is between 8%–20% (45–48). And the deletion or mutation in *FBW7* increases the protein level of c−Myc, which in turn increases the number of leukemia initiating cells. Furthermore, inhibition of the expression of c−Myc by small molecule inhibitors can inhibit the proliferation of mouse and human T-ALL cells, suggesting that *c−Myc* may be the proto-oncogene that drives T-ALL and is regulated by FBW7 (14). Besides, in T-ALL, *FBW7* mutation usually cooccurs with *Notch1* mutation, which has been widely proven to be an oncogene in T-ALL (49). Mutation of *FBW7* can reduce the cellular level of NICD protein degradation, which not only leads to abnormal activation of the Notch pathway but also reduces ubiquitination degradation of proto-oncogenes such as cyclin E and c-Myc (50). Kumar et al. demonstrated that Notch and NF-κB signaling can increase miR-223 gene expression, which in turn downregulates the expression of the onco-suppressor FBW7, known to negatively regulate Notch signaling, thus suggesting that the Notch/miR-223/FBW7 axis may reinforce Notch signaling effect in T-ALL. Furthermore, miR-223 may be involved in the mechanism of g-secretase inhibitor (GSI) treatment of patients with T-ALL; inhibiting miR-223 expression may reduce GSIs resistance (32). Moreover, *FBW7* mutations, region of R465C and R479Q, can also resist GSIs treatment by stabilizing NICD and its principle downstream target c-Myc, which may be the mechanism of GSIs resistance in patients with T-ALL (24). Moharram et al. showed the R465C mutation in *FBW7* played an important role in T-ALL progression when combined with Notch1 mutation and the R505C mutation in *FBW7* impaired Notch1 binding (46). In addition, approximately 60% of patients with T-ALL express the oncogenic transcription factor T-cell acute lymphocytic leukemia 1 (TAL1). Studies have shown that TAL1 can upregulate miR-223 expression by binding to the miR-223 promoter, reducing FBW7 expression, and increasing the expression of downstream substrates c-Myc, Notch1, and cyclin E, which ultimately induce cell proliferation (21, 26). Moreover, other studies also have shown that miR-223 can regulate T-ALL proliferation by reduce FBW7 expression; this indicates that miR-223 could regulate FBW7 activation in T-ALL (51, 52). Mcl-1 is an antiapoptotic protein of the BCL-2 family, which promotes tumor progression by inhibiting apoptosis (53). In T-ALL cell lines, the deletion of FBW7 increases in the expression level of Mcl-1 in a GSK3 phosphorylation-dependent manner and promotes T-ALL progression. Mcl-1 also upregulates resistance in the treatment of BCL-2 inhibitor (ABT737). However, when the FBW7 function was restored or Mcl-1 lost, the sensitivity to ABT737 was restored (19) (**Figure 1**).

However, other studies have shown that patients with *Notch1/FBW7* mutations have a better prognosis, with 5-year event-free survival of 95.5% and 5-year OS 100% (54). FBW7 deletion can upregulate glucocorticoid receptors in primary T-ALL cells, thereby enhancing their sensitivity to glucocorticoids and improving prognosis (55). In addition, *FBW7* mutations do not affect the prognosis of children with T-ALL. Maybe other oncogenes are involved or affected by treatment regimens. Therefore, more studies are still required to confirm the mechanisms related to the role of FBW7 in T-ALL.

### Adult T-cell leukemia/lymphoma

Adult T-cell leukemia/lymphoma (ATL) is a rare malignant T-cell monoclonal proliferative disease caused by human T-cell leukemia virus type 1 (HTLV1). Southwestern Japan is one of the most endemic areas for malignancy, along with the Caribbean basin, Central and South America. The Japan Clinical Oncology Group-Lymphoma Study Group proposed four clinical subtypes of ATL: acute, lymphoma, chronic, and smoldering. Nonetheless, the course of ATL is highly variable. Patients with acute, lymphoma, and chronic type with unfavorable prognostic factors, defined by levels of blood urea nitrogen or lactate dehydrogenase at higher than normal or having albumin levels lower than normal, are categorized as having aggressive clinical course; however, chronic type without unfavorable prognostic factors and smoldering type are indolent ATL (56, 57). *FBW7* is an important tumor suppressor, but some *FBW7* mutations can function as an oncogene. Yeh et al. showed that 25% (8/32) of patients with ATL had mutations in the *FBW7* WD40 domain, region of D510E and D527G, which had ability to target and degrade cyclin E, Mcl-1, and c-Myc. Mutation in *FBW7* failed to degrade NICD, activating the Notch signaling pathway and ultimately promoting ATL cell proliferation. However, FBW7 expression in wild-type, proliferation of cells was inhibited (25). In addition, c-Myc is a prognostic factor for patients with ATL, and FBW7 downregulation can lead to c-Myc accumulation and ATL cell proliferation (12). Patients with low FBW7 levels and high c-Myc levels experience poor prognosis, with 3-year OS less than 50% (12). Moreover, FBW7 mutations in ATL cells inhibit the degradation of BRAF and provide resistance to BET inhibitors through the RAF-MEK-ERK pathway, which also indicated ERK inhibitor may be the new therapeutic target in ATL (34). Therefore, FBW7 may be a potential target for the treatment of ATL (**Figure 1**).

## Proliferation of mature B lymphocytes

### Diffuse large B-cell lymphoma

DLBCL is the most common type of non-Hodgkin lymphoma in adults, accounting for 30%–40% of new diagnoses. DLBCL is classified as germinal center B-cell (GCB) subtype and activated B-cell (ABC) subtype by genetic profiling, and patients with the ABC subtype have significantly poorer outcomes (58). Approximately 60% of patients with DLBCL can cure with regimens such as rituximab, cyclophosphamide, adriamycin, vincristine, and prednisone (R-CHOP). However, 20%–50% of patients will be refractory or will relapse after achieving complete response (59). Therefore, ongoing efforts in the understanding of the mechanism of DLBCL have identified subsets of patients with poor prognosis for immunochemotherapy.

**Figure 2:**
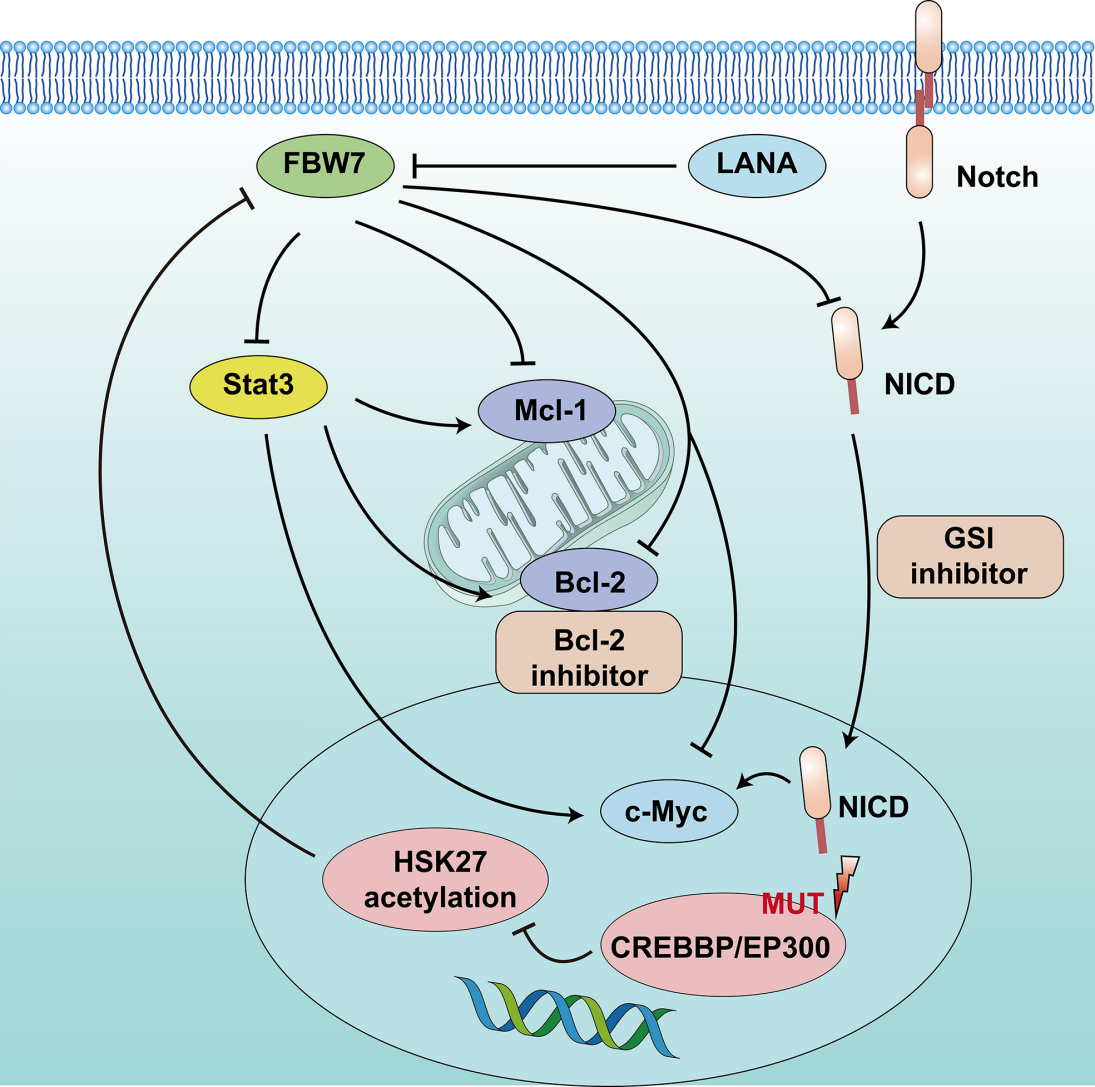
Schematic diagram of FBW7-mediated oncogene and signaling pathway in B lymphoproliferative malignancies. 1) CREBBP/EP300 mutations inhibit H3K27 acetylation, downregulate FBW7 expression, and activate the Notch pathway to promote cell proliferation. 2) Dysregulation of FBW7 could upregulate Stat3 expression and increase downstream antiapoptotic target genes, including c-Myc, Mcl-1, and Bcl-2, to induce cell proliferation and inhibit apoptosis.

FBW7 expression in patients with GCB-DLBCL was higher than in the ABC-DLBCL subtype, which had an inferior prognosis. The median survival time of patients in DLBCL with low FBW7 expression was 44 months, which was significantly shorter than that of those with high FBW7 expression (81 months) (22). FBW7 overexpression decreased cell viability and increased apoptosis rates in ABC-DLBCL cell lines. In terms of mechanism, stability of Stat3 and phospho-Stat3^Tyr705^ which are associated with poor survival in ABC-DLBCL, were reduced following FBW7 overexpression in ABC-DLBCL cell lines. The downstream antiapoptotic target genes of activated Stat3, including c-Myc, Mcl-1, Bcl-2, and Bcl-xl, showed decreased mRNA expression following exogenous FBW7 overexpression. Similarly, the negative relationship between FBW7 and Stat3 levels was also confirmed in DLBCL patient samples (22) (**Figure 2**).

In addition, epigenetic alterations play a vital role in the tumor progression of DLBCL. One study that adopted whole-genome/exome sequencing (WGS/WES) of 619 patients with DLBCL revealed that somatic mutations in *KMT2D* (19.5%) were most frequently observed, followed by mutations in *ARID1A* (8.7%), *CREBBP* (8.4%), *KMT2C* (8.2%), *TET2* (7.8%), *EP300* (6.8%), and *EZH2* (2.9%) (17). Somatic mutations in the *CREBBP* and *EP300* genes mainly occur in B-cell lymphomas, especially in DLBCL, and often relate to disease relapse and inferior prognosis. The 3-year progression-free survival and OS were 52.6% and 67.8%, respectively, lower than that of patients without mutation (17, 60). The mechanism suggested that *CREBBP/EP300* mutation inhibited H3K27 acetylation, downregulated FBW7 expression, and activated the Notch pathway, ultimately promoting DLBCL proliferation. This indicates that *CREBBP/EP300* mutation in DLBCL may regulate cell proliferation *via* the FBW7-Notch pathway (17) (**Figure 2**). However, another study reported that FBW7 was a tumor pro-survival factor (61). *KMT2D* is a tumor suppressor gene of DLBCL. FBW7 degrades KMT2D by ubiquitylation and promotes DLBCL cell growth. Therefore, more clinical studies are required to confirm the mechanisms related to the role of FBW7 in DLBCL.

### Chronic lymphocytic leukemia

CLL is a clonal proliferative disease of mature B lymphocytes, characterized by the aggregation of small mature lymphocytes in the peripheral blood, bone marrow, spleen, and lymph nodes. In addition, CLL is a heterogeneous disease with variable clinical presentation and evolution. CLL generally has a chronic and indolent course with slow progression. The treatment of watching and waiting is required at early stages, but CLL remains an incurable disease with heterogeneous prognosis. Patients with nonmutated IGHV, Del(17p), and Del(11q) chromosomal aberrations and *TP53* gene deletions or mutations usually have an aggressive course and poor prognosis and often do not achieve sustained remission (62, 63). Approximately 12% patients with CLL may have *Notch1* mutation, which is associated with poor prognosis (64). Patients of CLL with *Notch1* or *FBW7* mutations have a higher risk of Richter’s syndrome transformation (57%) (65). In addition, approximately 2%–8% of patients with CLL have detected *FBW7* mutations, mainly missense mutations, affecting the WD40 domain required for substrate binding, and the mutation sites detected at R465L, R465H and G423V (65–67). FBW7 negatively regulates Notch1. Close et al. used CRISPR/Cas9 technology to edit the WD40 domain in the CLL cell line to cause *FBW7* mutations, which could increase the transcription activity and protein level of Notch1 and c-Myc by reducing degradation (18). Moreover, *FBW7* mutations in CLL not only increase NICD levels but also increase the expression level of the Notch1 target gene, impeding its degradation through ubiquitination (18). These findings suggest that *FBW7* mutations play a role in activating the leukemia-causing Notch1 pathway (**Figure 2**).

### Primary effusion lymphoma

Primary effusion lymphoma (PEL) is a highly aggressive B-cell lymphoma with a poor prognosis and median survival of 6.2 months (68). The etiology is associated with Kaposi’s sarcoma associated herpesvirus (KSHV) (69). Latency-associated nuclear antigen (LANA) is a key gene of KSHV, which can interact with FBW7, resulting in inhibiting PEL cell apoptosis by reducing the degradation of Mcl-1 and caspase-3 through ubiquitination (29, 70) (**Figure 2**).

## Proliferation of bone marrow plasmacytes

### Multiple myeloma

MM is a hematologic malignancy characterized by the presence of abnormal clonal plasma cells in the bone marrow, accounting for 15% of adult hematologic malignancies. It is characterized by anemia, hypercalcemia, bone lesions, and kidney dysfunction. It remains an incurable disease, with a 5-year OS of approximately 45% (71–73). Therefore, understanding the underlying mechanisms of MM development is critical.

**Figure 3:**
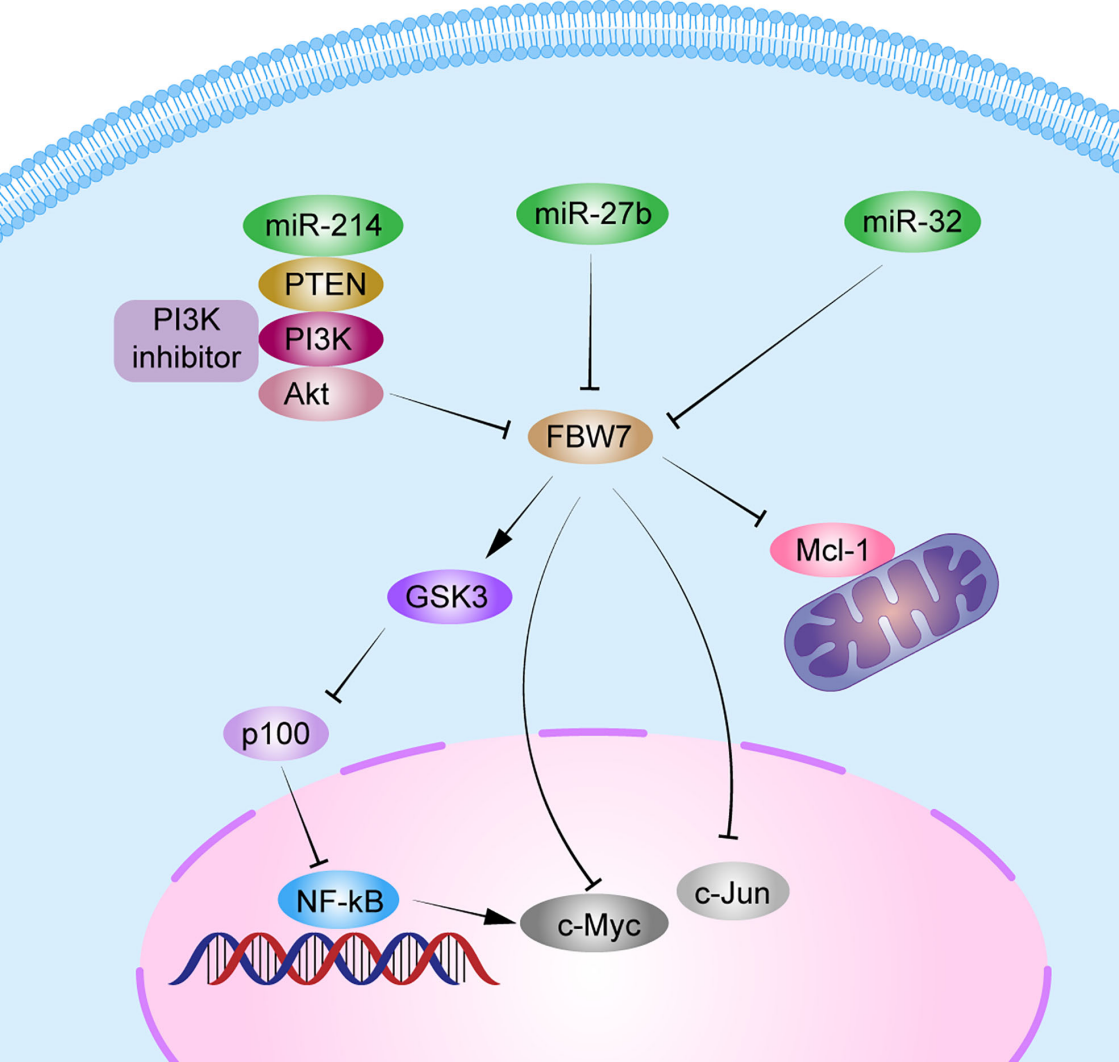
Schematic diagram of FBW7-mediated oncogene and signaling pathway in multiple myeloma. 1) FBW7 could target nuclear phosphorylation of p100 by GSK3β for degradation through ubiquitination, and subsequently activate the NF-κB pathway to promote MM cell proliferation. 2) miRNAs, such as miR-32, miR-27b, and miR-214, block degradation of Mcl-1, c-Myc, and c-Jun by FBW7 directly or indirectly by PTEN/AKT/GSK3 pathways to induce cell proliferation and apoptosis resistance.

The NF-κB pathway is important for cell growth, differentiation, and survival. It also plays a vital role in the development of MM (74). The p100 protein, belonging to the NF-family, is the main inhibitor of the noncanonical NF-κB pathway. Clearance of p100 from the nucleus is required for NF-κB pathway activation and MM cell survival. FBW7α can target the nuclear phosphorylation of p100 by GSK3 for degradation through ubiquitination both *in vivo* and *in vitro*. Thus, FBW7α and GSK3 function as pro-survival factors by controlling p100 degradation in MM (20, 75). Besides, miRNAs, such as miR-32, miR-27, miR-214, and miR-21, are upregulated in MM (76). Overexpression of miR-27b and miR-214 in MM can mediate the FBW7 and PTEN/AKT/GSK3 pathways blocking degradation by ubiquitinating Mcl-1 and inducing cell proliferation and apoptosis resistance (30). In addition, patients with MM with a lower FBW7 expression and higher miR-32 expression in cancer tissues than in normal tissues, had poorer prognosis, with a median OS <3 years. Overexpression of miR-32 *in vitro* decreased FBW7 expression and increased the expression of cancer-related proteins, c-Jun and c-Myc (23). These results indicate that miRNAs can regulate FBW7 expression in MM and then affect the expression of its downstream substrates, thus promoting MM proliferation. In all, *FBW7* can be both a tumor suppressor gene and a pro-survival gene in MM. Further studies should confirm the mechanisms related to the role of FBW7 in MM (**Figure 3**).

### Other malignant lymphoproliferative disorders

Mutations of the *FBW7* gene were also detected in B-ALL and mantle cell lymphoma (77–79). MiR-27a is upregulated in pediatric B-ALL, and its expression is inversely correlated with FBW7 expression and disease progression (80). Further studies are required to clarify this mechanism. In addition, FBW7 overexpression in cutaneous T-cell lymphoma could increase the sensitivity of histone deacetylase inhibitor (HDACi) (81).

## Conclusion

FBW7 as a critical tumor suppressor, can recognize various oncogenic proteins and degrade them through ubiquitination to maintain cell growth. However, FBW7 dysregulation is associated with the development of malignant lymphoproliferative disorders. In addition, *FBW7* mutations or inactivation is associated with chemoresistance and poor prognosis. Thus, the underlying mechanisms can potentially be targeted for treating malignant lymphoproliferative disorders. Future studies should attempt to elucidate the complex mechanisms underlying the role of FBW7 and its substrates and to identify novel targets for effective treatment of malignant lymphoproliferative disorders. Clinical studies are needed to confirm the significance of FBW7 in tumor development, progression, and resistance to therapies as well as opportunities for targeted therapies.

## Author contributions

XW, WG, ZZ and OB designed the study and contributed vital data and analytical tools and wrote the manuscript. All authors contributed to the article and approved the submitted version.

## Funding

This study was funded by the Science and Technology Agency of Jilin province (20200201591JC).

## Conflict of interest

The authors declare that the research was conducted in the absence of any commercial or financial relationships that could be construed as a potential conflict of interest.

## Publisher’s note

All claims expressed in this article are solely those of the authors and do not necessarily represent those of their affiliated organizations, or those of the publisher, the editors and the reviewers. Any product that may be evaluated in this article, or claim that may be made by its manufacturer, is not guaranteed or endorsed by the publisher.

## References

[1] Blombery PA Dickinson M Westerman DA. Molecular lesions in b-cell lymphoproliferative disorders: Recent contributions from studies utilizing high-throughput sequencing techniques. Leuk Lymphoma (2014) 55(1):19–30. 23550993 10.3109/10428194.2013.792112

[2] Yumimoto K Nakayama KI. Recent insight into the role of FBXW7 as a tumor suppressor. Semin Cancer Biol (2020) 67(Pt 2):1–15. 10.1016/j.semcancer.2020.02.017 32113998

[3] Oberg C Li J Pauley A Wolf E Gurney M Lendahl U. The notch intracellular domain is ubiquitinated and negatively regulated by the mammalian sel-10 homolog. J Biol Chem (2001) 276(38):35847–53. 10.1074/jbc.M103992200 11461910

[4] Shimizu K Nihira NT Inuzuka H Wei W. Physiological functions of FBW7 in cancer and metabolism. Cell Signal (2018) 46:15–22. 29474981 10.1016/j.cellsig.2018.02.009 PMC5882551

[5] Zhu Q Hu L Guo Y Xiao Z Xu Q Tong X. FBW7 in hematological tumors. Oncol Lett (2020) 19(3):1657–64. 10.3892/ol.2020.11264 PMC7039162 32194657

[6] Ye Z Zhuo Q Hu Q Xu X Mengqi L Zhang Z. FBW7-NRA41-SCD1 axis synchronously regulates apoptosis and ferroptosis in pancreatic cancer cells. Redox Biol (2021) 38:101807. 33271455 10.1016/j.redox.2020.101807 PMC7710650

[7] Xu F Li J Ni M Cheng J Zhao H Wang S. FBW7 suppresses ovarian cancer development by targeting the N(6)-methyladenosine binding protein YTHDF2. Mol Cancer (2021) 20(1):45. 33658012 10.1186/s12943-021-01340-8 PMC7927415

[8] Kim MJ Chen G Sica GL Deng X. Epigenetic modulation of FBW7/Mcl-1 pathway for lung cancer therapy. Cancer Biol Ther (2021) 22(1):55–65. 33336620 10.1080/15384047.2020.1856756 PMC7833779

[9] Li Q Li Y Li J Ma Y Dai W Mo S. FBW7 suppresses metastasis of colorectal cancer by inhibiting HIF1α/CEACAM5 functional axis. Int J Biol Sci (2018) 14(7):726–35. 10.7150/ijbs.24505 PMC6001674 29910683

[10] Yeh CH Bellon M Nicot C. FBXW7: A critical tumor suppressor of human cancers. Mol Cancer (2018) 17(1):115. 30086763 10.1186/s12943-018-0857-2 PMC6081812

[11] Chen J Ding C Chen Y Hu W Lu Y Wu W. ACSL4 promotes hepatocellular carcinoma progression *via* c-myc stability mediated by ERK/FBW7/c-myc axis. Oncogenesis (2020) 9(4):42. 32350243 10.1038/s41389-020-0226-z PMC7190855

[12] Mihashi Y Mizoguchi M Takamatsu Y Ishitsuka K Iwasaki H Koga M. C-MYC and its main ubiquitin ligase, FBXW7, influence cell proliferation and prognosis in adult T-cell Leukemia/Lymphoma. Am J Surg Pathol (2017) 41(8):1139–49. 10.1097/PAS.0000000000000871 28498285

[13] Zhang Z Liu M Hu Q Xu W Liu W Sun Q. FGFBP1, a downstream target of the FBW7/c-myc axis, promotes cell proliferation and migration in pancreatic cancer. Am J Cancer Res (2019) 9(12):2650–64. PMC6943353 31911852

[14] King B Trimarchi T Reavie L Xu L Mullenders J Ntziachristos P. The ubiquitin ligase FBXW7 modulates leukemia-initiating cell activity by regulating MYC stability. Cell (2013) 153(7):1552–66. 10.1016/j.cell.2013.05.041 PMC4146439 23791182

[15] Davis RJ Swanger J Hughes BT Clurman BE. The PP2A-B56 phosphatase opposes cyclin e autocatalytic degradation *via* site-specific dephosphorylation. Mol Cell Biol (2017) 37(8):1–13. 10.1128/MCB.00657-16 PMC5376636 28137908

[16] Tosello V Ferrando AA. The NOTCH signaling pathway: role in the pathogenesis of T-cell acute lymphoblastic leukemia and implication for therapy. Ther Adv Hematol (2013) 4(3):199–210. 23730497 10.1177/2040620712471368 PMC3666445

[17] Huang YH Cai K Xu PP Wang L Huang CX Fang Y. CREBBP/EP300 mutations promoted tumor progression in diffuse large b-cell lymphoma through altering tumor-associated macrophage polarization *via* FBXW7-NOTCH-CCL2/CSF1 axis. Signal Transduct Target Ther (2021) 6(1):10. 33431788 10.1038/s41392-020-00437-8 PMC7801454

[18] Close V Close W Kugler SJ Reichenzeller M Yosifov DY Bloehdorn J. FBXW7 mutations reduce binding of NOTCH1, leading to cleaved NOTCH1 accumulation and target gene activation in CLL. Blood (2019) 133(8):830–9. 10.1182/blood-2018-09-874529 30510140

[19] Inuzuka H Shaik S Onoyama I Gao D Tseng A Maser RS. SCF(FBW7) regulates cellular apoptosis by targeting MCL1 for ubiquitylation and destruction. Nature (2011) 471(7336):104–9. 10.1038/nature09732 PMC3076007 21368833

[20] Busino L Millman SE Pagano M. SCF-mediated degradation of p100 (NF-κB2): mechanisms and relevance in multiple myeloma. Sci Signal (2012) 5(253):pt14. 23211527 10.1126/scisignal.2003408 PMC3871187

[21] Vázquez-Domínguez I González-Sánchez L López-Nieva P Fernández-Navarro P Villa-Morales M Cobos-Fernández M. Downregulation of specific FBXW7 isoforms with differential effects in T-cell lymphoblastic lymphoma. Oncogene (2019) 38(23):4620–36. 10.1038/s41388-019-0746-1 30742097

[22] Yao S Xu F Chen Y Ge Y Zhang F Huang H. Fbw7 regulates apoptosis in activated b-cell like diffuse large b-cell lymphoma by targeting Stat3 for ubiquitylation and degradation. J Exp Clin Cancer Res (2017) 36(1):10. 28069035 10.1186/s13046-016-0476-y PMC5223361

[23] Hua J Ding T Yang L. Dysfunction of microRNA-32 regulates ubiquitin ligase FBXW7 in multiple myeloma disease. Onco Targets Ther (2016) 9:6573–9. 10.2147/OTT.S105945 PMC5087813 27822062

[24] O’Neil J Grim J Strack P Rao S Tibbitts D Winter C. FBW7 mutations in leukemic cells mediate NOTCH pathway activation and resistance to gamma-secretase inhibitors. J Exp Med (2007) 204(8):1813–24. 10.1084/jem.20070876 PMC2118656 17646409

[25] Yeh CH Bellon M Pancewicz-Wojtkiewicz J Nicot C. Oncogenic mutations in the FBXW7 gene of adult T-cell leukemia patients. Proc Natl Acad Sci U S A (2016) 113(24):6731–6. 10.1073/pnas.1601537113 PMC4914202 27247421

[26] Mansour MR Sanda T Lawton LN Li X Kreslavsky T Novina CD. The TAL1 complex targets the FBXW7 tumor suppressor by activating miR-223 in human T cell acute lymphoblastic leukemia. J Exp Med (2013) 210(8):1545–57. 10.1084/jem.20122516 PMC3727321 23857984

[27] Ye X Nalepa G Welcker M Kessler BM Spooner E Qin J. Recognition of phosphodegron motifs in human cyclin e by the SCF(Fbw7) ubiquitin ligase. J Biol Chem (2004) 279(48):50110–9. 10.1074/jbc.M409226200 15364936

[28] Wei W Jin J Schlisio S Harper JW Kaelin WG Jr.. The v-jun point mutation allows c-jun to escape GSK3-dependent recognition and destruction by the Fbw7 ubiquitin ligase. Cancer Cell (2005) 8(1):25–33. 16023596 10.1016/j.ccr.2005.06.005

[29] Kim YJ Kim Y Kumar A Kim CW Toth Z Cho NH. Kaposi’s sarcoma-associated herpesvirus latency-associated nuclear antigen dysregulates expression of MCL-1 by targeting FBW7. PloS Pathog (2021) 17(1):e1009179. 33471866 10.1371/journal.ppat.1009179 PMC7816990

[30] Frassanito MA Desantis V Di Marzo L Craparotta I Beltrame L Marchini S. Bone marrow fibroblasts overexpress miR-27b and miR-214 in step with multiple myeloma progression, dependent on tumour cell-derived exosomes. J Pathol (2019) 247(2):241–53. 10.1002/path.5187 30357841

[31] Gao F Yu X Li M Zhou L Liu W Li W. Deguelin suppresses non-small cell lung cancer by inhibiting EGFR signaling and promoting GSK3β/FBW7-mediated mcl-1 destabilization. Cell Death Dis (2020) 11(2):143. 32081857 10.1038/s41419-020-2344-0 PMC7035355

[32] Kumar V Palermo R Talora C Campese AF Checquolo S Bellavia D. Notch and NF-kB signaling pathways regulate miR-223/FBXW7 axis in T-cell acute lymphoblastic leukemia. Leukemia (2014) 28(12):2324–35. 10.1038/leu.2014.133 24727676

[33] Fukushima H Matsumoto A Inuzuka H Zhai B Lau AW Wan L. SCF(Fbw7) modulates the NFkB signaling pathway by targeting NFkB2 for ubiquitination and destruction. Cell Rep (2012) 1(5):434–43. 10.1016/j.celrep.2012.04.002 PMC3375724 22708077

[34] Yeh CH Bellon M Wang F Zhang H Fu L Nicot C. Loss of FBXW7-mediated degradation of BRAF elicits resistance to BET inhibitors in adult T cell leukemia cells. Mol Cancer (2020) 19(1):139. 32907612 10.1186/s12943-020-01254-x PMC7487643

[35] Sailo BL Banik K Girisa S Bordoloi D Fan L Halim CE. FBXW7 in cancer: What has been unraveled thus far? Cancers (Basel) (2019) 11(2):1–31. 10.3390/cancers11020246 PMC6406609 30791487

[36] Matsuoka S Oike Y Onoyama I Iwama A Arai F Takubo K. Fbxw7 acts as a critical fail-safe against premature loss of hematopoietic stem cells and development of T-ALL. Genes Dev (2008) 22(8):986–91. 10.1101/gad.1621808 PMC2335330 18367647

[37] Balamurugan K Mendoza-Villanueva D Sharan S Summers GH Dobrolecki LE Lewis MT. C/EBPδ links IL-6 and HIF-1 signaling to promote breast cancer stem cell-associated phenotypes. Oncogene (2019) 38(20):3765–80. 10.1038/s41388-018-0516-5 PMC6437025 30262865

[38] Pui CH Robison LL Look AT. Acute lymphoblastic leukaemia. Lancet (2008) 371(9617):1030–43. 10.1016/S0140-6736(08)60457-2 18358930

[39] Brown P Inaba H Annesley C Beck J Colace S Dallas M. Pediatric acute lymphoblastic leukemia, version 2.2020, NCCN clinical practice guidelines in oncology. J Natl Compr Canc Netw (2020) 18(1):81–112. 31910389 10.6004/jnccn.2020.0001

[40] O’Brien S Thomas D Ravandi F Faderl S Cortes J Borthakur G. Outcome of adults with acute lymphocytic leukemia after second salvage therapy. Cancer (2008) 113(11):3186–91. 10.1002/cncr.23919 PMC4188532 18846563

[41] Bhanushali AA Babu S Thangapandi VR Pillai R Chheda P Das BR. Mutations in the HD and PEST domain of notch-1 receptor in T-cell acute lymphoblastic leukemia: report of novel mutations from Indian population. Oncol Res (2010) 19(2):99–104. 21302811 10.3727/096504010x12864748215007

[42] Espinosa L Cathelin S D’Altri T Trimarchi T Statnikov A Guiu J. The Notch/Hes1 pathway sustains NF-κB activation through CYLD repression in T cell leukemia. Cancer Cell (2010) 18(3):268–81. 10.1016/j.ccr.2010.08.006 PMC2963042 20832754

[43] Wallaert A Durinck K Taghon T Van Vlierberghe P Speleman F. T-ALL. And thymocytes: a message of noncoding RNAs. J Hematol Oncol (2017) 10(1):66. 28270163 10.1186/s13045-017-0432-0 PMC5341419

[44] Onoyama I Tsunematsu R Matsumoto A Kimura T de Alborán IM Nakayama K. Conditional inactivation of Fbxw7 impairs cell-cycle exit during T cell differentiation and results in lymphomatogenesis. J Exp Med (2007) 204(12):2875–88. 10.1084/jem.20062299 PMC2118521 17984302

[45] Yuan L Lu L Yang Y Sun H Chen X Huang Y. Genetic mutational profiling analysis of T cell acute lymphoblastic leukemia reveal mutant FBXW7 as a prognostic indicator for inferior survival. Ann Hematol (2015) 94(11):1817–28. 10.1007/s00277-015-2474-0 26341754

[46] Moharram SA Shah K Kazi JU. T-Cell acute lymphoblastic leukemia cells display activation of different survival pathways. J Cancer (2017) 8(19):4124. 29187889 10.7150/jca.21725 PMC5706016

[47] Baldus CD Thibaut J Goekbuget N Stroux A Schlee C Mossner M. Prognostic implications of NOTCH1 and FBXW7 mutations in adult acute T-lymphoblastic leukemia. Haematologica (2009) 94(10):1383–90. 10.3324/haematol.2008.005272 PMC2754954 19794083

[48] Kraszewska MD Dawidowska M Kosmalska M Sędek L Grzeszczak W Kowalczyk JR. BCL11B, FLT3, NOTCH1 and FBXW7 mutation status in T-cell acute lymphoblastic leukemia patients. Blood Cells Mol Dis (2013) 50(1):33–8. 10.1016/j.bcmd.2012.09.001 23040356

[49] Thompson BJ Jankovic V Gao J Buonamici S Vest A Lee JM. Control of hematopoietic stem cell quiescence by the E3 ubiquitin ligase Fbw7. J Exp Med (2008) 205(6):1395–408. 10.1084/jem.20080277 PMC2413036 18474632

[50] Thompson BJ Buonamici S Sulis ML Palomero T Vilimas T Basso G. The SCFFBW7 ubiquitin ligase complex as a tumor suppressor in T cell leukemia. J Exp Med (2007) 204(8):1825–35. 10.1084/jem.20070872 PMC2118676 17646408

[51] Hou Y Sun J Huang J Yao F Chen X Zhu B. Correction to: Circular RNA circRNA_0000094 sponges microRNA−223−3p and up−regulate f−box and WD repeat domain containing 7 to restrain T cell acute lymphoblastic leukemia progression. Hum Cell (2021) 34(5):1584. 33677796 10.1007/s13577-021-00504-4

[52] Mavrakis KJ van der Meulen J Wolfe AL Liu X Mets E Taghon T. A cooperative microRNA-tumor suppressor gene network in acute T-cell lymphoblastic leukemia (T-ALL). Nat Genet (2011) 43(7):673–8. 10.1038/ng.858 PMC4121855 21642990

[53] Sancho M Leiva D Lucendo E Orzáez M. Understanding MCL1: From cellular function and regulation to pharmacological inhibition. FEBS J (2021) 289(20):6209–34. 10.1111/febs.16136 PMC9787394 34310025

[54] Park MJ Taki T Oda M Watanabe T Yumura-Yagi K Kobayashi R. FBXW7 and NOTCH1 mutations in childhood T cell acute lymphoblastic leukaemia and T cell non-Hodgkin lymphoma. Br J Haematol (2009) 145(2):198–206. 19245433 10.1111/j.1365-2141.2009.07607.x

[55] Malyukova A Brown S Papa R O’Brien R Giles J Trahair TN. FBXW7 regulates glucocorticoid response in T-cell acute lymphoblastic leukaemia by targeting the glucocorticoid receptor for degradation. Leukemia (2013) 27(5):1053–62. 10.1038/leu.2012.361 23228967

[56] Katsuya H Ishitsuka K Utsunomiya A Hanada S Eto T Moriuchi Y. Treatment and survival among 1594 patients with ATL. Blood (2015) 126(24):2570–7. 10.1182/blood-2015-03-632489 26361794

[57] Ishitsuka K Tamura K. Human T-cell leukaemia virus type I and adult T-cell leukaemia-lymphoma. Lancet Oncol (2014) 15(11):e517–e26. 10.1016/S1470-2045(14)70202-5 25281470

[58] Susanibar-Adaniya S Barta SK. 2021 Update on diffuse large b cell lymphoma: A review of current data and potential applications on risk stratification and management. Am J Hematol (2021) 96(5):617–29. 10.1002/ajh.26151 PMC8172085 33661537

[59] Crump M Neelapu SS Farooq U Van Den Neste E Kuruvilla J Westin J. Outcomes in refractory diffuse large b-cell lymphoma: results from the international SCHOLAR-1 study. Blood (2017) 130(16):1800–8. 10.1182/blood-2017-03-769620 PMC5649550 28774879

[60] Juskevicius D Jucker D Klingbiel D Mamot C Dirnhofer S Tzankov A. Mutations of CREBBP and SOCS1 are independent prognostic factors in diffuse large b cell lymphoma: Mutational analysis of the SAKK 38/07 prospective clinical trial cohort. J Hematol Oncol (2017) 10(1):70. 28302137 10.1186/s13045-017-0438-7 PMC5356266

[61] Saffie R Zhou N Rolland D Önder Ö Basrur V Campbell S. FBXW7 triggers degradation of KMT2D to favor growth of diffuse Large b-cell lymphoma cells. Cancer Res (2020) 80(12):2498–511. 10.1158/0008-5472.CAN-19-2247 PMC7417195 32350066

[62] Hallek M Al-Sawaf O. Chronic lymphocytic leukemia: 2022 update on diagnostic and therapeutic procedures. Am J Hematol (2021) 96(12):1679–705. 10.1002/ajh.26367 34625994

[63] Quinquenel A Aurran-Schleinitz T Clavert A Cymbalista F Dartigeas C Davi F. Diagnosis and treatment of chronic lymphocytic leukemia: Recommendations of the French CLL study group (FILO). Hemasphere (2020) 4(5):e473. 33062946 10.1097/HS9.0000000000000473 PMC7523785

[64] Puente XS Pinyol M Quesada V Conde L Ordóñez GR Villamor N. Whole-genome sequencing identifies recurrent mutations in chronic lymphocytic leukaemia. Nature (2011) 475(7354):101–5. 10.1038/nature10113 PMC3322590 21642962

[65] Falisi E Novella E Visco C Guercini N Maura F Giaretta I. B-cell receptor configuration and mutational analysis of patients with chronic lymphocytic leukaemia and trisomy 12 reveal recurrent molecular abnormalities. Hematol Oncol (2014) 32(1):22–30. 23861036 10.1002/hon.2086

[66] Landau DA Tausch E Taylor-Weiner AN Stewart C Reiter JG Bahlo J. Mutations driving CLL and their evolution in progression and relapse. Nature (2015) 526(7574):525–30. 10.1038/nature15395 PMC4815041 26466571

[67] Quijada-Álamo M Hernández-Sánchez M Robledo C Hernández-Sánchez JM Benito R Montaño A. Next-generation sequencing and FISH studies reveal the appearance of gene mutations and chromosomal abnormalities in hematopoietic progenitors in chronic lymphocytic leukemia. J Hematol Oncol (2017) 10(1):83. 28399885 10.1186/s13045-017-0450-y PMC5387353

[68] Boulanger E Gérard L Gabarre J Molina JM Rapp C Abino JF. Prognostic factors and outcome of human herpesvirus 8-associated primary effusion lymphoma in patients with AIDS. J Clin Oncol (2005) 23(19):4372–80. 10.1200/JCO.2005.07.084 15994147

[69] Gonçalves PH Uldrick TS Yarchoan R. HIV-Associated kaposi sarcoma and related diseases. Aids (2017) 31(14):1903–16. 10.1097/QAD.0000000000001567 PMC6310482 28609402

[70] Juillard F Tan M Li S Kaye KM. Kaposi’s sarcoma herpesvirus genome persistence. Front Microbiol (2016) 7:1149. 27570517 10.3389/fmicb.2016.01149 PMC4982378

[71] Kazandjian D. Multiple myeloma epidemiology and survival: A unique malignancy. Semin Oncol (2016) 43(6):676–81. 10.1053/j.seminoncol.2016.11.004 PMC5283695 28061985

[72] Kumar SK Rajkumar V Kyle RA van Duin M Sonneveld P Mateos MV. Multiple myeloma. Nat Rev Dis Primers (2017) 3:17046. 28726797 10.1038/nrdp.2017.46

[73] Cowan AJ Green DJ Kwok M Lee S Coffey DG Holmberg LA. Diagnosis and management of multiple myeloma: A review. JAMA (2022) 327(5):464–77. 10.1001/jama.2022.0003 35103762

[74] Dehghanifard A Kaviani S Abroun S Mehdizadeh M Saiedi S Maali A. Various signaling pathways in multiple myeloma cells and effects of treatment on these pathways. Clin Lymphoma Myeloma Leuk (2018) 18(5):311–20. 10.1016/j.clml.2018.03.007 29606369

[75] Busino L Millman SE Scotto L Kyratsous CA Basrur V O’Connor O. Fbxw7α- and GSK3-mediated degradation of p100 is a pro-survival mechanism in multiple myeloma. Nat Cell Biol (2012) 14(4):375–85. 10.1038/ncb2463 PMC3339029 22388891

[76] Chen D Yang X Liu M Zhang Z Xing E. Roles of miRNA dysregulation in the pathogenesis of multiple myeloma. Cancer Gene Ther (2021) 28(12):1256–68. 10.1038/s41417-020-00291-4 PMC8636266 33402729

[77] Song JH Schnittke N Zaat A Walsh CS Miller CW. FBXW7 mutation in adult T-cell and b-cell acute lymphocytic leukemias. Leuk Res (2008) 32(11):1751–5. 10.1016/j.leukres.2008.03.040 18485478

[78] Sakhdari A Ok CY Patel KP Kanagal-Shamanna R Yin CC Zuo Z. TP53 mutations are common in mantle cell lymphoma, including the indolent leukemic non-nodal variant. Ann Diagn Pathol (2019) 41:38–42. 31132650 10.1016/j.anndiagpath.2019.05.004

[79] Jiang P Desai A Ye H. Progress in molecular feature of smoldering mantle cell lymphoma. Exp Hematol Oncol (2021) 10(1):41. 34256839 10.1186/s40164-021-00232-3 PMC8278675

[80] Lerner M Lundgren J Akhoondi S Jahn A Ng HF Akbari Moqadam F. MiRNA-27a controls FBW7/hCDC4-dependent cyclin e degradation and cell cycle progression. Cell Cycle (2011) 10(13):2172–83. 10.4161/cc.10.13.16248 21597324

[81] Napoli M Venkatanarayan A Raulji P Meyers BA Norton W Mangala LS. ΔNp63/DGCR8-dependent MicroRNAs mediate therapeutic efficacy of HDAC inhibitors in cancer. Cancer Cell (2016) 29(6):874–88. 10.1016/j.ccell.2016.04.016 PMC4908836 27300436

